# Post-overdose follow-up in the community with peer recovery specialists: The Lake Superior Diversion and Substance Use Response Team

**DOI:** 10.1016/j.dadr.2023.100139

**Published:** 2023-02-01

**Authors:** Bradley Ray, Jessica McCarthy-Nickila, Nicholas Richardson, Jeffrey Maahs

**Affiliations:** a RTI International, 3040 Cornwallis Road, Research Triangle Park, NC 27709, USA; b Duluth Police Department, 2030N Arlington Ave, Duluth, MN 55811, USA; c University of Minnesota Duluth, Studies in Justice, Culture, & Social Change, 1123 University Drive, Duluth, MN 55812, USA

## Abstract

**Background:**

As the overdose epidemic continues to worsen, nonfatal overdose calls for service represent a critical touchpoint for intervention. While most studies have focused on law enforcement led post-overdose follow-up, the current study describes the programmatic characteristics and outcomes of a non-law enforcement post overdose program comprised of peer specialists embedded within a local police department.

**Methods:**

We examined information on 341 follow-up responses occurring over a 16-month study period using administrative data. We assessed programmatic characteristics including demographic information on clients, referral source, engagement type, and goal completion.

**Results:**

The results indicate that over 60% of client referrals ended in the goal of in-person contact. Of those, about 80% went on to complete an engagement goal with the peer specialist. We found no significant variation in client demographics and referral source or follow-up engagement (in-person or not); however, client referrals from law enforcement first responders, the most common source, are significantly less likely to result in an in-person contact, though, if contact is made, similarly likely to complete an engagement goal.

**Conclusions:**

Post overdose response programs that do not involve law enforcement are exceedingly rare. Given that some research has shown that police involvement in post overdose response can have unexpected, associated harms, it is important to assess the effectiveness of post overdose programs that do not involve the police. Findings here suggest that this type of program is successful at locating and engaging community members into recovery support services who have experienced an overdose.

## Highlights

• Peer-led post-overdose response program embedded in police department.
• Analysis of 16-months of administrative program data on 341 clients.
• Over 60% of referrals resulted in in-person contact.
• Over 80% completed an engagement goal with peer specialists.

## 1 Introduction

The illicit drug market has continued to transform over the past two decades in ways that contribute to escalating overdose rates globally (Volkow et al., 2019). In the United States, nearly one million died from a drug overdose over the past twenty years. Most of these overdose deaths were opioid-related, though the type of opioid has varied across multiple waves, each resulting in more deaths (Ahmad et al., 2022). Since 2013, increases in overdose deaths are primarily the result of illicitly manufactured fentanyl, a synthetic opioid 50 times more potent than heroin (Ciccarone, 2021). Fortunately, opioid-related overdoses can be reversed through administration of naloxone, an antagonist that reverses respiratory depression caused by opioids. In most jurisdictions emergency medical services (EMS) are deployed to all poisoning events, regardless of the substance type, and have been equipped with naloxone since the 1970s (Sternbach et al., 1980). As the overdose epidemic has accelerated, it has become common practice to equip other first responders, particularly law enforcement, with naloxone. In many jurisdictions, law enforcement respond to all poisoning events and sometimes are first to arrive on scene (Pourtaher et al., 2022; Pozo, 2022). Research suggests law enforcement can effectively administer naloxone (Fisher et al., 2016; Rando et al., 2015); however, there may also be drawbacks as police regularly confiscate personal belongings, force overdose survivors to go to the hospital, and make it more likely that an overdose event results in an arrest (Lowder et al., 2020; Ray et al., 2022; Smiley-McDonald et al., 2022).

From a public health perspective, nonfatal overdose represents a critical touchpoint for intervention (Larochelle et al., 2019) to reduce risk of a fatal overdose. Indeed, hospitals in particular appear well positioned to play a crucial role; however, some patients refuse transport to the hospital after a nonfatal overdose (Ray et al., 2020; Wampler et al., 2011), or do not find a hospital setting conducive for starting recovery (Pollini et al., 2006), and not all jurisdictions transport overdose victims to the hospital as there remains no established standard of care for persons post overdose. In response, many communities have developed multi-agency collaborations to provide a follow-up response to community members after a nonfatal overdose with the goal of referral into community-based treatment and services. Although there appears to be great variability in response efforts, research has focused almost exclusively on law enforcement driven responses.

National prevalence of post-overdose follow-up efforts suggests most programs include law enforcement during the follow-up, most often with EMS (Ray et al., 2023). Research on these efforts has been limited primarily to case studies and state-wide surveys with findings that suggests little consistency in the practices, partners, or services provided and largely focused on efforts led by, or that include sworn law enforcement in the follow-up response with little rigorous evaluation of effectiveness (Canada and Formica, 2022; Davoust et al., 2021; Donnelly et al., 2022; Formica et al., 2018, 2022; Tori et al., 2022; Wagner et al., 2016). Important concerns have been raised about post-overdose response involving law enforcement, from unintended effects through increased mistrust of social services (Doe-Simkins et al., 2022; Latimore and Bergstein, 2017; van der Meulen et al., 2021; Wagner et al., 2021) to specific activities like “warrant checking,” prior to outreach that might ultimately undermine program goals (Tori et al., 2022). Law enforcement presence after an overdose could lead to a negative impact on willingness to engage in follow-up or accept assistance as research with overdose survivors suggests concerns about law enforcement involvement in post-overdose interventions resulting in further criminal legal entanglement (Wagner et al., 2019).

In this short report we describe such a program that is facilitated by peer recovery specialists. Importantly, the team is diverse in terms of substance use background (e.g., stimulants and opioids), recovery experience, and race-ethnicity such that they are representative of the community they serve. We describe the characteristics of this program in detail and provide evidence of feasibility by exploring client characteristics and service engagement efforts to begin developing an evidence-base around post-overdose response efforts that address concerns expressed among those with lived nonfatal overdose experiences.

### 1.1 Setting and methods

In February 2019, The Lake Superior Diversion and Substance Use Response Team (SURT) started working directly with clients following non-fatal overdose. The team is embedded within the Duluth Police Department which employs approximately 150 sworn officers. Duluth is in St. Louis County, Minnesota with a population of 86,700 in the city limits and 290,000 in the metro area which includes Superior, Wisconsin (Douglas County). While the overdose rates in Minnesota are generally lower than other US states, the overdose rates in St. Louis County are consistently higher than the state average and have closely mirrored the national trends, increasing dramatically over the past two decades over multiple waves shifting from prescription opioids, to heroin, and finally to illicitly manufactured fentanyl.

The SURT consists of four peer recovery specialists and a licensed social worker (LSW) with alcohol and drug counseling certification, all of whom have physical workspace within the police department and are employed as civilian staff. The LSW is supervised by a contracted Licensed Independent Clinical Social Worker from the local drug and alcohol treatment center and three of peers are supervised are supervised by the SURT project manager, who is also a certified peer specialist, and who reports to law enforcement personnel. They coordinate regularly with an incident analyst (also non-sworn civilian staff), whose role is to identify overdose follow-up opportunities in the community. The incident analyst maintains a database with information from overdose calls for service where police were present. Peer specialists use this database to facilitate outreach through phone, social media (e.g., messaging or chatting), and in-person follow-up to either the individuals home address (if known) or the address where the overdose event occurred. As the SURT became established, peer specialists also cultivated direct law enforcement referrals (e.g., a text or email from a responding officer), as well as referrals from community members and agencies. Regardless of referral source, SURT maintains a secured database, unavailable to others in the agency, that confidentially tracks client contact information.

In this short report, we are unable to assess program effectiveness but instead examine SURT administrative records to provide evidence of feasibility by exploring client characteristics and service engagement efforts. While the program peers have been working directly with clients since 2019, the present study focuses on data from 341 cases over 16-months (March 1, 2021 through June 30, 2022) that were collected once a SURT records management system was established. Importantly, we were not able to access information on all overdose calls for service in the area during this period so we cannot establish a rate of overall referral but instead use all cases identified by the incident analyst and provide to the SURT peers as our denominator (*N* = 341) to look at the rate and duration of follow-up. Records management also includes a measure of contact hours which includes efforts to contact the person and follow-up peer support which encompass time spent identifying available treatment and waiting at appointments. All data were collected as part of routine practice and deidentified for analysis as part of a program evaluation and non-human participant research. Variables were coded from administrative data with descriptive and bivariate analysis (Chi-square and t-tests) conducted in SPSS (version 26; SPSS, Armok, NY).

## 2 Results

During the 16-month study period, there were 341 unique referrals, an average of more than 20 per month. Nearly two-thirds (62.5%) were male, and the average age was 35.9 years. More than one-quarter were Indigenous or American Native persons (26.7%) and more than two-thirds were white (68.1%). Referrals came from a variety of sources with the most (43.7%) from law enforcement first responders, 24.9% from other criminal-legal agencies including courts and community supervision; and 16.1% through self-referral (Table 1).

**Table 1:** Client demographics, referral source, and follow-up engagement (*N* = 341).

		Mean	SD
Age (years)		35.85	11.26
*Race-Ethnicity Categories*		N	Percent
	American Indian/Alaska Native	88	26.7%
	Asian	1	0.3%
	Black/African American	14	4.3%
	Hispanic/Latino	2	0.6%
	White/Caucasian	224	68.1%
*Gender*			
	Female	128	37.5%
	Male	213	62.5%
*Referral Source*			
	Law enforcement first responder	149	43.7%
	Criminal-legal agency	85	24.9%
	Community stakeholder	26	7.6%
	Self-referral	55	16.1%
	Family or friend	26	7.6%
*Engagement Type*			
	In-person contact	207	60.7%
	No contact	134	39.3%
*Any completed goals (n* *=* *207)*			
	Yes	166	80.2%
	No	41	19.8%

Notes: *N* = 341. March 1, 2021, through June 30, 2022. Age missing for 22 cases.

In-person contact is the goal of the follow-up, and was accomplished with 60.7% of referrals (i.e., SURT had in-person contact with 207 of 341 persons who were identified as having had an overdose), of which 80.2% went on to complete an engagement goal with the peer specialist. There was an average of 2 engagement goals per client, which resulted from an average of 4.6 contact hours (SD=6.9), with assessments (34.8%) as the most common goal followed by treatment engagement (28.6%) and entry into a detox facility (19.0%) (Table 2). There was no significant variation in client demographics (age, race-ethnicity, gender), nor referral source or follow-up engagement (in-person or not); however, client referrals from law enforcement first responders at nonfatal overdose events are significantly less likely to result in an in-person contact (38.3% vs. 78.1%, *χ*2 = 55.9, Cramer's *V* = 0.405, *p*<.001) though, if contact was made, are statistically similar in likelihood to complete an engagement goal than those from other referral sources (75.4% and 82.0%, respectively).

**Table 2:** Follow-up engagement outcomes.

		Mean	SD
Number of goals completed		2.0	1.5
Number of contact hours		4.6	6.9
*Goal category frequency*		N	Percent
	Assessment	117	34.8%
	Education	2	0.6%
	Employment	7	2.1%
	Detox facility	64	19.0%
	Enter/Start treatment	96	28.6%
	Health care	6	1.8%
	Housing	15	4.5%
	Recovery supportive services	29	8.6%

Notes: March 1, 2021, through June 30, 2022.

## 3 Discussion

In this short report we described and examined administrative records from a novel and innovative post-overdose follow-up response program led by peer recovery specialists who are embedded with the local city police department. Given that all of the published research to-date has focused on law enforcement led post-overdose follow-up, we present our findings as evidence of an alternative approach communities might consider. Peer recovery specialists are a paraprofessional workforce with an emerging evidence-base on the positive effects of incorporating these persons of lived experience as part of the recovery support process, particularly among those who are navigating criminal-legal systems involvement but also in overdose response efforts (Bardwell et al., 2018; Bartlett et al., 2011; Felton et al., 2023; Victor et al., 2021).

While there are documented barriers to hiring peer specialists, specifically because of prior involvement in criminal-legal systems, the SURT sees this experience as an advantage as it gives peer staff shared experience with the people they serve. Given that more than one-quarter of clients were American Indian/Alaskan Native, this same philosophy guided SURT in bringing on a Native American male to focus on identifying effective means of engagement within this shared cultural context. The SURT peer specialists have autonomy over their work and leverage their embeddedness in a criminal-legal system to build trust by helping clients to address outstanding warrants and avoid arrest, sometimes by engaging directly with community supervision and agents of the court, to mitigate negative legal consequences. Moreover, with employment as civilian staff within the police department, the peer specialists are provided a more competitive salary, along with benefits that come from being a city employee (e.g., pension), which can assist with recruiting diverse applicants, job retention, and long-term program sustainability.

The SURT peer specialists recognize the stages of behavior change (DiClemente, 1999) and much of their initial outreach is focused on developing rapport and determining needed areas of recovery support. They also provide a range of harm reduction support services including fentanyl testing strips, naloxone, new syringes, and infectious disease testing. From initial outreach through rapport building it is unlikely SURT would be as successful if police were present. It is difficult to compare given the variation in programs and study designs but existing research suggests police and EMS post overdose response programs respectively range from 54% (Formica et al., 2018) to 60% (Scharf et al., 2021) in terms of contact where SURT was able to achieve in-person contact with 60.7% of referrals, of which 80% engaged with peer specialists to complete a recovery-oriented goal. However, much further research is needed to assess program effectiveness and determine the rate of referral from all overdose calls for service in the study jurisdiction. Additionally, it is a notable finding that law enforcement referrals were less likely to result in an in-person contact, and while SURT peers would suggest it results from less appropriate or difficult to contact referrals (e.g., unsheltered persons who cannot be located in the community), future research should aim to understand which nonfatal overdose survivors are in greatest need and most appropriate for peer-led follow up services. The present study is also limited to the administrative data fields, which were not designed for research purposes; as such, a primary goal for future research should focus on developing a comparison group and assessing changes in treatment engagement, criminal-legal systems involvement, emergency medical events, and mortality following SURT. Additionally, contact hours does not capture the nuance in SURT activity but instead a measure of overall time spent with the client including contact efforts. Moreover, the data collection period started approximately one-year after COVID-19 stay-at-home orders in the state and so referrals and follow-up before or after might differ based on this factor. Despite the limitations, the findings offer an important contribution to the research literature by documenting outcomes from a novel-overdose response program of peer specialists who are embedded within a local law enforcement agency.

## 4 Conclusion

At a time when communities are questioning the role of police in the overdose epidemic, it is important to note that unannounced follow-up from law enforcement might be exacerbating overdose-related harms. Even well-intentioned officers might induce fear and trauma among overdose survivors and their family. Yet, nonfatal overdose does provide a critical opportunity to link people with health systems and social supports. The model described in this report is not driven by a law enforcement agency, but is facilitated through it, and run by persons with lived experience in recovery. The findings here suggest that this post-overdose response program is successful at locating and engaging community members who have had an overdose into recovery support services.

## Contributors

Bradley Ray, Jessica McCarthy-Nickila, Nicholas Richardson, and Jeffrey Maahs. BR conceived of manuscript and wrote initial draft and incorporated feedback from JMN, NR, and JM. JM developed program data collection, JMN oversaw program and reviewed for accuracy of description, NR assisted with analysis and literature review. All authors contributed to the final written manuscript.

## Role of funding source

This work was supported by the 10.13039/100005196Bureau of Justice Assistance’s Comprehensive Opioid, Stimulant, and Substance Abuse Program (2019-AR-BX-K053). They did not have any role in study design, data collection, analysis and interpretation of data, the writing of the report, or the decision to submit the article for publication.

## Declaration of Competing Interest

No authors have any conflicts to declare but note that McCarthy-Nickila (co-author) was employed by the Duluth Police Department and oversaw program activities during the reported study period.
